# Gap Expansion Between Cranial Bone and Polyether Ether Ketone Implant in Cranioplasty of Pediatric Patients after Decompressive Craniectomy at Follow-up

**DOI:** 10.1089/neur.2025.0007

**Published:** 2025-08-11

**Authors:** Chuanwei Wang, Chen Yang, Runlu Zhang, Yuan Zhang, Yanzhao Wang, Liping Ning, Guoran Du, Zhaoxi Sang, Shilei Ni, Xingang Li, Jie Gong

**Affiliations:** ^1^ Department of Neurosurgery, Qilu Hospital, Cheeloo College of Medicine and Institute of Brain and Brain-Inspired Science, Shandong University, Jinan, China.; ^2^ Shandong Key Laboratory of Brain Health and Function Remodeling, Jinan, China.; ^3^ Clinical Epidemiology Unit, Qilu Hospital of Shandong University, Jinan, China.; ^4^ Rehabilitation Department, Shandong Provincial Hospital Affiliated to Shandong First Medical University, Jinan, China.; ^5^ Department of Neurosurgery, Qingyun People’s Hospital, Dezhou, China.; ^6^ Department of Neurosurgery, People’s Hospital of Chiping District, Liaocheng, China.

**Keywords:** adolescence, children, cranioplasty, pediatrics, polyether ether ketone

## Abstract

This study aimed to explore the experience and complications of cranioplasty (CP) with polyether ether ketone (PEEK) in pediatric and adolescent patients after decompressive craniectomy (DC). A total of 62 children (aged <18 years) with cranial bone defects due to DC underwent CP with a custom-made PEEK at our department between January 2018 and April 2023. The clinical characteristics, radiological features, surgical conditions, postoperative complications, and follow-up results of these patients were analyzed retrospectively. Kaplan–Meier survival analysis and Cox regression were used to analyze data. The age of the patients ranged from 2 to 17 years. The follow-up periods ranged from 12 to 70 months. Six patients experienced subcutaneous fluid accumulation (9.7%), five experienced epidural fluid accumulation (8.1%), and two experienced scalp inflammation (3.2%), which all were cured before discharge. Seven patients experienced bone gap expansion at the interface between the cranial bone and PEEK during follow-up (11.3%). Univariate analysis showed that DC-CP time interval (<3 months) and age were two influencing factors. Multivariate analysis revealed that age was the most important factor (*p* < 0.005, hazard ratio = 0.250, 95% confidence interval: 0.096–0.652). No reoperation was performed. Medical follow-ups were carried out further. For pediatric patients with cranial defects after DC who receive CP with a custom-made PEEK, two variables including younger age and too short DC-CP time interval may be unfavorable factors, to make patients experience bone gap expansion at the interface between the cranial bone and the PEEK. Additional data should be collected to validate our conclusions.

## Introduction

Craniocerebral trauma ranks first among all organ injuries in children and adolescents in terms of mortality and disability rates.^1^ Decompressive craniectomy (DC) is an effective treatment for patients with high intracranial pressure that is refractory to medicine, which may be caused by head trauma, spontaneous cerebral hemorrhage, or cerebral infarction. Patients entering the recovery period after DC face issues such as lack of brain protection and cosmetic problems caused by skull defects. Cranial bone defect reconstruction, also known as cranioplasty (CP), can address problems with autologous bone or artificial materials. Many studies have been conducted on skull defect repair in adults and children, and an international consensus has been reached.^2^ However, the skulls of children are still in the growth and development stages, and bony hyperplasia and absorption may occur more frequently in children than in adults. Several issues on CP, such as the optimal time, best implant, and common complications, remain unaddressed in pediatric patients. Many reconstruction materials can be used, each of which has its own advantages and disadvantages. Other diseases, such as congenital anomalies, calvarium tumors, and osteofibrous dysplasia, also require bone reconstruction after mass resection. In this study, we only elucidate CP with a custom-made polyether ether ketone (PEEK) in pediatric patients with cranial defects after DC.

## Materials and Methods

The study was conducted with the approval of the Ethics Committee, Qilu Hospital of Shandong University (KYLL-202312[YJ]-017). All procedures complied with the principles laid down in the Declaration of Helsinki. All written informed consent forms were got from the patients’ guardians. Sixty-two patients (<18 years) who had cranial bone defects due to DC several months or years ago underwent CP with custom-made PEEK implant at the Neurosurgery Department, Qilu Hospital of Shandong University, between January 2018 and April 2023. Inclusion criteria included (1) age (0–18 years); (2) sex (male and female); (3) reason of cranial defect (DC); and (4) CP using PEEK. Exclusion criteria included (1) patients having severe comorbidities such as organ dysfunctions, blood abnormalities, or infection and (2) patients or their guardians who did not have compliance with medical follow-up or medical research.

A high-resolution computed tomography (CT) of the head was performed to generate accurate data to create a custom-made PEEK implant (Kontour, Xi^,^an, China) with the help of computer-aided design and manufacturing. The same skilled neurosurgical teams performed all CP surgeries. All patients received intravenous administration of prophylactic antibiotics half an hour before skin incision. Continuous percutaneous drainage was placed in all patients and removed 2 or 3 days after surgery. The following data were analyzed: clinical characteristics, radiological features, PEEK parameters, surgical conditions, postoperative complications, and follow-up results. A total of seven cases had a gap expansion at the interface between the cranial bone margin and PEEK at follow-up, which we called “bone gap expansion.” We selected this interesting phenomenon as the key research object. Survival time was defined as the time from operation to gap expansion at the interface between the cranial bone margin and PEEK. Univariate analysis was employed to screen factors influencing bone gap expansion using Kaplan–Meier survival analysis. Multivariate Cox regression analysis was performed to analyze the most vital factor based on the univariate analysis results. SPSS 20.0 was used to analyze data, and statistical significance was set at a *p*-value <0.05.

## Results

This study included 18 females and 44 males. There was a predominance of men in these patients. Patients’ ages ranged from 2 to 17 years (median age 7.0 years). The time interval between DC and CP (abbreviated as DC-CP time interval) ranged from 1 to 108 months. The maximum diameter of the PEEK implant ranged from 6 to 14 cm. The actual distance between the bone defect margin and the midline (abbreviated as the distance to the midline) varied from 0 to 7.4 cm. Preoperative radiological images showed intracranial comorbidities such as encephalomalacia, in cases with no hydrocephalus. Prophylactic antibiotics included cefazolin, cefuroxime, ceftriaxone, and vancomycin in 13, 18, 27, and 4 patients, respectively. Operative time varied from 1.5 to 4 h. There were no severe complications such as new-onset neurological dysfunction, intracranial hemorrhage, or death in this series. Postoperatively, six patients experienced subcutaneous fluid accumulation (9.7%), five experienced epidural fluid accumulation (8.1%), and two experienced scalp inflammation (3.2%), all of which were cured or improved before discharge.

**FIG. 1.**
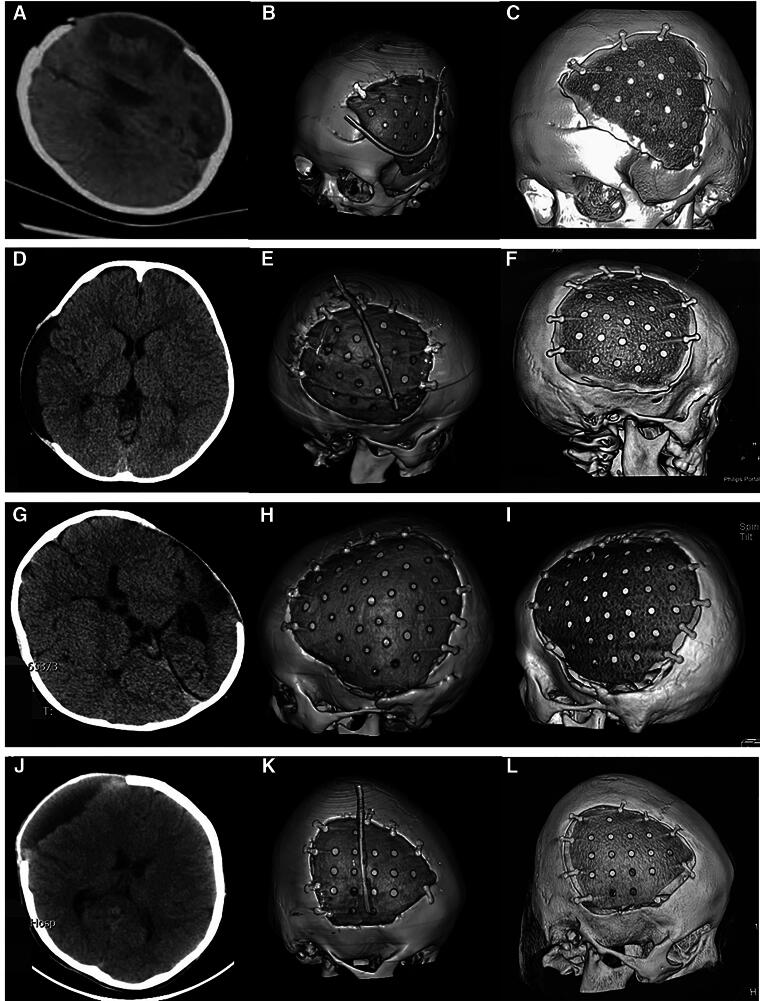
CT images of four typical patients with bone gap expansion at the interface between cranial bone and PEEK. **(A, D, G, J)** Preoperative CT image. **(B, E, H, K)** Postoperative 3D CT image. **(C, F, I, L)** Follow-up 3D CT image. Patient 1: A, B, C; patient 2: D, E, F; patient 3: G, H, I; patient 4: J, K, L. CT, computed tomography; PEEK, polyether ether ketone.

**FIG. 2.**
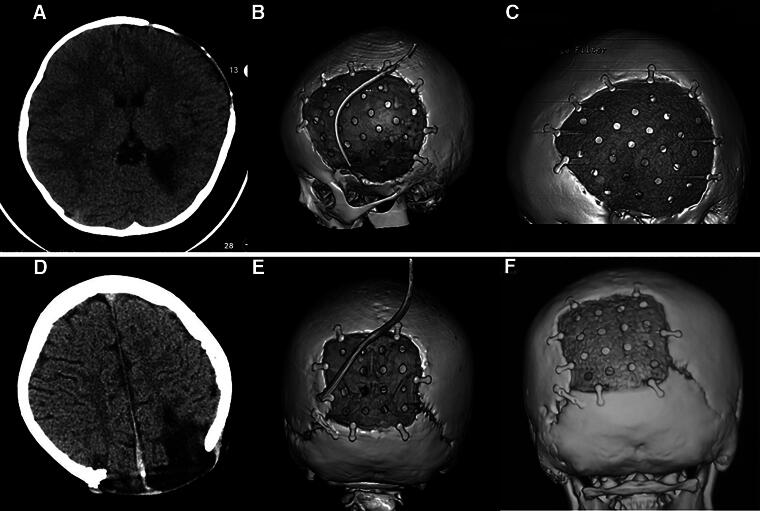
CT images of two typical patients with no bone gap expansion at the interface between cranial bone and PEEK. **(A, D)** Preoperative CT image. **(B, E)** Postoperative 3D CT image. **(C, F)** Follow-up 3D CT image. Patient 1: A, B, C; patient 2: D, E, F. CT, computed tomography; PEEK, polyether ether ketone.

**FIG. 3.**
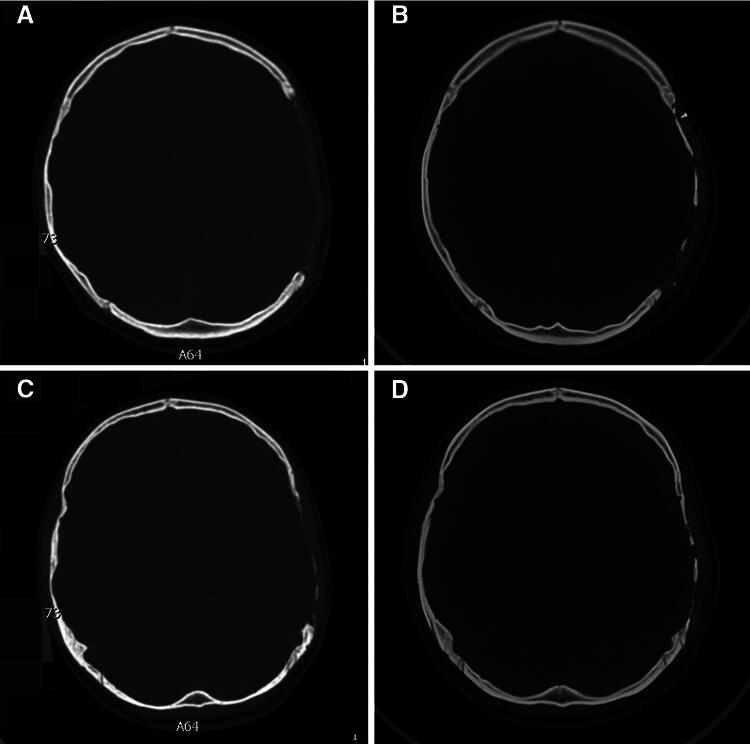
CT images of two typical patients with osteoconduction in the outer layer of the dura mater below the PEEK implant. **(A, C)** Preoperative CT image. **(B, D)** Follow-up CT image. Patient 1: A, B; patient 2: C, D. CT, computed tomography; PEEK, polyether ether ketone.

The follow-up periods ranged from 12 to 70 months (median: 31 months). None were lost to follow-up. Seven patients experienced bone gap expansion at the interface between the cranial bone and PEEK implant at follow-up (11.3%). CT images of typical four patients are shown in Figure 1. The other cases showed good performance in the CT images (Fig. 2). The appearance of all patients was good. Several factors were analyzed, and the results are listed in Table 1. Kaplan–Meier survival analysis showed that too short DC-CP time interval (<3 months) and younger age were two influencing factors (Table 1). Cox regression revealed that age was the most important factor, which means that more younger children may experience bone gap expansion at the interface between the cranial bone and PEEK than older children at follow-up (Table 2). Three patients exhibited osteoconduction in the outer layer of dura mater below the PEEK implant (Fig. 3).

**Table 1.** Univariate Analysis of Factors on Bone Gap Expansion at the Interface Between Cranial Bone and Polyether Ether Ketone

Variable	Number	Event	*p*
Age (years)			0.007
<4	11	4	
4–6	18	3	
7–10	13	0	
>10	20	0	
Sex			0.425
Male	44	6	
Female	18	1	
DC-CP interval (months)			0.003
<3	20	7	
3–6	24	0	
7–12	6	0	
>12	12	0	
Scalp complication			0.875
No	48	6	
Local effusion	11	1	
Cellulitis or infection	3	0	
Maximum diameter (cm)			0.463
6–10	20	3	
>10	42	4	
Distance to midline (cm)			0.425
0–1	9	0	
2–4	6	2	
5–6	12	1	
>6	35	4	
Preoperative encephalomalacia			0.124
Yes	37	1	
No	25	6	
Crossing cranial sutures			0.288
Yes	53	7	
No	9	0	
Sides			0.432
Left	29	5	
Right	29	2	
Bilateral	4	0	

Maximum diameter: maximum diameter of bone defect; distance to midline: distance between the bone defect margin to midline; crossing cranial suture: bone defect crossing cranial suture.

CP, cranioplasty; DC, decompressive craniectomy; DC-CP interval: time interval between DC and CP.

**Table 2.** Cox Regression Analysis of Factors on Bone Gap Expansion at the Interface Between Cranial Bone and Polyether Ether Ketone

Variable	Wald	Sig	Exp(B)	95% CI	
				Down	Up
Age	8.036	0.005	0.250	0.096	0.652

CI, confidence interval.

## Discussion

CP is the gold standard treatment for patients with cranial defects after DC. This procedure was first used thousands of years ago.^3^ The main goal of CP is to protect the brain and restore esthetics in patients with cranial defects. It is also helpful in relieving neurological and psychosocial symptoms, which are deemed to be due to improved cerebrospinal fluid dynamics and cerebral blood flow.^4–6^ CP is more beneficial in children. First, children are active and have poor self-control and a decreased awareness of danger, leading to a higher risk of re-trauma to brain tissue due to a lack of skull protection. Second, almost half of the children who undergo DC due to traumatic brain injury have cognitive abnormalities, some of which are related to skull defects.^5,6^ These patients may exhibit improvement clinical manifestations after CP.

At present, an ideal material for CP in patients with cranial defects, especially in children, remains controversial.^2,7–10^ The autologous bone remains the cornerstone of cranial defect repair because of its biocompatibility. Preserved autologous bone has been the gold standard for many years. However, although there are some new technologies, such as biomaterials, tissue engineering, and stem cell delivery, the problem of bone resorption has not yet been solved for preserved autologous bone flaps. The incidence of bone flap absorption in children is higher than that in adults: 100% in infants, 80% in children under 8 years, and 50% in the general pediatric population.^3,10–13^ One meta-analysis showed that CP performed with fresh, autologous, heterotopic cranial bone grafts brings lower complication rate compared with alloplastic materials, but the object of this study is adult.^14^ Harvesting autografts from other donor sites such as the rib or iliac crest, an autologous bone reconstruction method, seems to be an alternative for older children but may be not a good option for younger children because of insufficient donor bones and high complication rates.^3,10^ A systematic review including 10,346 CPs in all age-groups showed a higher removal rate of autologous bone than that of alloplastic materials.^11^

Many artificial materials have been developed for alloplastic CP, such as PEEK, titanium mesh, polymethyl methacrylate (PMMA), hydroxyapatite (HA), and nano-hap/collagen composites.^2,7–9,15,16^ However, these materials have advantages as well as disadvantages. The ideal features of allografts for cranial repair should be mechanical resistance, nonconductivity of heat, cold and electricity, ease of use, and even osteoconductivity and biodegradability.^7–9,16^ Custom-made alloplastic implants made of titanium, PEEK, or PMMA can provide a good esthetic appearance. Although titanium mesh is strong and covers the edge of the cranial defect, it is heat- and electricity-conductive and induces large artifacts in CT images.^17,18^ HA is similar to the bone structure and can offer osteoconduction and osteogenesis but is also fragile. Many children, especially younger ones, are sometimes active and boisterous; therefore, secondary damage is more prevalent in children than in adults, which can lead to the deformation of the titanium mesh or cracks in the HA. PEEK has the following advantages: mechanical resistance, light weight, ease of use, nonconductivity of heat, cold and electricity, nonmagnetic, and translucent to X-rays. PEEK can be used as alternatives for repairing cranial defects in children.

However, additional requirements must be met for alloplastic CP in children. For example, allografts should coordinate cranial growth or bone absorption in children. Unfortunately, no artificial material currently meets this criterion.^3,8,9,16^ In this study, postoperative osteoconduction was found in three patients during the follow-up period, and no harmful effects on these patients were observed; therefore, no treatments were administered, but medical follow-up was continued. However, it was surprising that bone gap expansion at the interface between the cranial bone and the PEEK occurred in seven patients at follow-up. Fortunately, until the end of the follow-up period, most of the titanium screws and plates still fixed the bones; therefore, no re-operation was performed, and medical follow-up continues. We will closely follow up these patients because problems, such as hardware loosening of the PEEK implants, may occur in the future.

To the best of our knowledge, this is the first report with a notable number of pediatric patients experiencing bone gap expansion at the interface between the cranial bone and the PEEK implant at follow-up. To explore the reasons, we collected data on several variables that could potentially influence the event. We found that two factors including “younger age” and “short DC-CP time interval” may play a vital role. The results were based on Kaplan–Meier survival analysis. To diminish the bias of the univariate analysis, a subsequent multivariate analysis via the Cox regression model was used, which revealed that younger age was the most critical factor. In cranial bones, intramembranous bone growth is achieved within the periosteum or by bone formation at the sutures.^19^ Cranial sutures and the periosteum maintain normal craniofacial development in young children, allow skull movement, and create a reservoir of mesenchymal stem cells and osteoblasts responsible for bone growth. Head circumference may reach 80% of its adult size by 2 years and 90% by 5 years.^20,21^ The skull grows minimally after 6 years. In our data, seven patients with “bone gap expansion” included one case (2 years old), three cases (3 years old), one case (4 years old), and two cases (5 years old) (details can be seen in Fig. 1 and Table 3), all of whom were less than 6 years old. Therefore, we assume that the periosteum might not be intact and that bone sutures were broken after DC in some patients, impairing the bone growth ability. Meanwhile, for young children with cranial defects after DC, head circumference is enlarged during cranial development, but the bone edge cannot produce meaningful bone tissue. Although there have been a few cases of postoperative osteoconduction, these newly formed bones cannot grow into the older cranial bone. Consequently, the bone gap at the interface between the cranial bone and the PEEK expands consistently with the growing skull, similar to a theory in the literature.^10^ PEEK implants cannot coordinate with neurocranium growth. Other studies showed cases and attributed this phenomenon to bone resorption at the interface in pediatric CP.^3,22^ Considering that an excessively short DC-CP time interval may induce expansion of the bone gap, we hypothesize that resorption of the bone edge may occur in early stages after surgery, which means pediatric patients who received CP immediately after DC (<3 months) may experience fast bone resorption adjacent to the margin of the bone defects. However, this hypothesis needs to be verified in additional cases.

**Table 3.** Data of Seven Cases with Bone Gap Expansion at the Interface Between Cranial Bone and Polyether Ether Ketone

Patient no.	Sex	Age (years)	DC-CP (months)	Max diam (cm)	Dis to mid (cm)	Side	Cro cra suture	Post infla	Follow-up time (months)
1	M	4	2	13.5	7.0	L	Y	N	38
2	M	2	2	9.5	5.0	L	Y	Y	25
3	M	3	1	9.5	4.0	R	Y	N	24
4	M	5	2	12.0	7.0	L	Y	N	12
5	F	3	2	13.0	8.0	R	Y	N	36
6	M	3	1	9.5	4.0	R	Y	N	24
7	M	5	2	12.0	7.0	L	Y	N	16

Cro cra suture: bone defect crossing cranial sutures; Dis to mid: distance between the bone defect margin to midline; Max diam: maximum diameter of bone defect; Post infla: postoperative inflammation.

CP, cranioplasty; DC, decompressive craniectomy; DC-CP: time interval between DC and CP.

Based on our contemporary techniques, one strategy may be used to deal with this difficulty of PEEK CP in younger pediatric patients. We have already used an increasing number of longer titanium or PEEK plates and titanium screws to fix the PEEK and bone closely together, to reduce the pulling force in every plate, and to reduce the rate of plates falling off the PEEK and bones. Additional cases should be investigated to confirm the advantages and disadvantages of the method. Although not all autologous bones and alloplastic implants are perfect, younger children still require cranial repair when their physical condition is stable owing to a higher risk of re-trauma to the brain tissue after DC in the pediatric population. We expect better materials than the currently used material to be developed for cranial reconstruction in the future.

Other variables, including sex, scalp complications, maximum diameter of the bone defects (maximum diameter), distance between the bone defects margin and the midline (distance to midline), preoperative encephalomalacia, bone defects crossing the cranial sutures (crossing the cranial sutures), and sides (right, left, and both), were considered not statistically significant. Therefore, if we use PEEK reconstruction in children with bone defects after DC, more attention should be paid to age and the DC-CP time interval rather than the diameter of the bone defects or other factors.

Two cases involved postoperative scalp inflammation that were cured before discharge. We believe that strict handwashing procedures, double gloving, prophylactic antibiotics, and surgical field washing with normal saline may help reduce the infection rate after CP. Blood supply to the scalp should be maintained, and the use of an electrosurgical pencil should be minimized.

Limitations of the study include: it was not a multicenter research; it was a retrospective study; the number of cases was not big enough; and this research was only about PEEK implant, not being referring to other materials such as autologous implant or titanium implant.

## Conclusions

For pediatric patients with cranial defects after DC who receive CP with a custom-made PEEK, two variables including younger age and too short DC-CP time interval may be unfavorable factors, to make patients experience bone gap expansion at the interface between the cranial bone and the PEEK. Additional data should be collected to validate our conclusions.

## Transparency, Rigor and Reproducibility Summary

The study was pre-registered at https://www.medicalresearch.org.cn/login (MR-37-24-037438). The analysis plan was registered after beginning data collection but before data analysis at https://www.medicalresearch.org.cn/login (MR-37-24-037438). A sample size of 62 subjects was planned based on availability of subjects. There were 62 participants in whom primary clinical outcomes were assessed. Participants were told the results of their prognostic assessments. Data collection was performed by investigators who were aware of relevant participant characteristics. Data analyses were performed by investigators blinded to relevant characteristics of the participants. All data used to develop prognostic models are available from the authors. The key inclusion criteria and outcome evaluations are established standards. Replication by the study group was performed as part of this study. De-identified data from this study are not available in a public archive. De-identified data from this study will be made available (as allowable according to institutional review board standards) by emailing the corresponding authors. Analytic codes used to conduct the analyses presented in this study are not available in a public repository. They may be available by emailing the corresponding authors. The authors agree to publish the article using the Mary Ann Liebert Inc. “Open Access” option under appropriate license.

## Authors’ Contributions

Conception or design of the work: C.W., C.Y., R.Z., S.N., X.L., and J.G. Acquisition of data for the work: C.W., C.Y., R.Z., Y.W., L.N., G.D., and Z.S. Analysis and interpretation of data for the work: C.W., C.Y., R.Z., S.N., X.L., and J.G. Drafting the work: C.W., C.Y., and R.Z. Reviewing it critically for important intellectual content: C.W., X.L, and J.G. Final approval of the version to be published: C.W., C.Y., R.Z., Y.W., L.N., G.D., Z.S., S.N., X.L, and J.G. Agreement to be accountable for all aspects of the work in ensuring that questions related to the accuracy or integrity of any part of the work are appropriately investigated and resolved: C.W., C.Y., R.Z., Y.W., L.N., G.D., Z.S., S.N., X.L., and J.G.

## Abbreviations Used

CI: confidence interval
CP: cranioplasty
CT: computed tomography
DC: decompressive craniectomy
HA: hydroxyapatite
MRI: magnetic resonance imaging
PEEK: polyether ether ketone
PMMA: polymethyl methacrylate
WHO: World Health Organization
